# Need for analgesia after percutaneous liver biopsy: a real-life experience

**DOI:** 10.1590/0100-3984.2020.0035

**Published:** 2021

**Authors:** Ricardo Holderbaum do Amaral, Fabrice C. Deprez, João Pedro Dalla-Bona, Guilherme Watte, Rômulo Santos Roxo, Edson Marchiori, Bruno Hochhegger

**Affiliations:** 1 Laboratório de Pesquisa em Imagens Médicas (Labimed) da Universidade Federal de Ciências da Saúde de Porto Alegre (UFCSPA), Porto Alegre, RS, Brazil.; 2 Cliniques Universitaires UCL de Mont-Godinne, Yvoir, Belgium.; 3 Universidade Federal do Rio de Janeiro (UFRJ), Rio de Janeiro, RJ, Brazil.

**Keywords:** Liver diseases, Ultrasonography, Radiology, interventional, Biopsy, needle, Analgesia, Pain management, Hepatopatias, Ultrassonografia, Radiologia intervencionista, Biópsia por agulha, Analgesia, Manejo da dor

## Abstract

**Objective:**

To evaluate variables affecting the need for analgesia after ultrasound-guided percutaneous liver biopsy performed on an outpatient basis.

**Materials and Methods:**

This was a retrospective analysis of 1,042 liver biopsies performed between 2012 and 2018. The data collected included the age and sex of the patient, as well as self-reported pain in the recovery room, the pain treatment used, the indication for the biopsy, and the lobe punctured. As per the protocol of our institution, physicians would re-evaluate patients with mild pain (1-3 on a visual analog scale), prescribe analgesics for those with moderate pain (4-6 on the visual analog scale), and prescribe opioids for those with severe pain (7-10 on the visual analog scale).

**Results:**

The main indications for biopsy were related to diffuse disease (in 89.9%), including the follow-up of hepatitis C (in 47.0%) and suspicion of nonalcoholic steatohepatitis (in 38.0%). Pain requiring analgesia occurred in 8.0% of procedures. Of the 485 female patients, 51 (10.5%) needed analgesia, compared with 33 (5.9%) of the 557 male patients (*p* < 0.05). The need for analgesia did not differ in relation to patient age, the lobe punctured, or the indication for biopsy (nodular or diffuse disease). The analgesic most commonly used was dipyrone (in 75.9%), followed by paracetamol alone (16.4%) and their combination with opioids (7.6%).

**Conclusion:**

Ultrasound-guided percutaneous liver biopsy is safe and well tolerated. Postprocedural pain does not correlate with the lobe punctured, patient age, or the indication for biopsy and appears to affect more women than men.

## Abstract (pt)

**Objetivo:**

Avaliar variáveis que afetam a necessidade de analgesia após biópsia hepática guiada por ultrassonografia.

**Materiais e Métodos:**

Análise retrospectiva de 1042 biópsias hepáticas realizadas entre 2012 e 2018. Os dados coletados incluíram dor detectada na sala de recuperação, analgesia utilizada, indicação, lobo puncionado, idade e sexo do paciente. O protocolo institucional indicava orientações e reavaliação para dor leve (1-3, segundo a escala visual analógica), analgésicos simples para dor moderada (4-6, segundo a escala visual analógica) e opioides para dor importante (7-10, segundo a escala visual analógica).

**Resultados:**

As indicações foram principalmente doença difusa (89,9%), particularmente no seguimento de hepatite C (47,0%) e suspeita de esteato-hepatite não alcoólica (38,0%). Dor com necessidade de analgesia ocorreu em 8,0% dos procedimentos. Mulheres demandaram analgesia em 10,5% das vezes e homens demandaram em 5,9% (*p* < 0,05). Não houve diferença estatisticamente significante na necessidade de analgesia em relação a idade, lobo hepático puncionado ou indicação por doença nodular *versus* difusa. O analgésico mais utilizado foi dipirona (75,9%), seguido de paracetamol (16,4%) e associação com opioides (7,6%).

**Conclusão:**

Este é um procedimento seguro e bem tolerado. Dor pós-procedimento não se correlaciona com lateralidade da biópsia, idade ou doença nodular *versus* difusa e parece afetar mais mulheres que homens.

## INTRODUCTION

Liver biopsy plays an important role in the evaluation of liver disease. Direct tissue examination is key for the confirmation and grading of chronic hepatitis, allowing fibrosis to be staged, as well as allowing associated diseases and alternative diagnoses to be excluded^(1-4)^. Despite the development of new techniques for cirrhosis staging, such as elastography, liver biopsy continues to be the classical gold-standard technique^(2,5-7)^. For focal lesions or masses, a tissue core obtained through biopsy for histological analysis provides optimal conditions for further tissue processing, such as a more refined immunohistochemical analysis^(8,9)^. The current methods for obtaining liver tissue samples are percutaneous, transjugular, endoscopic and laparoscopic biopsy^(3,10-12)^. Among the percutaneous methods, the most widely used are sheathed (Tru-Cut) needle biopsy, suction (Menghini) needle biopsy, and, less commonly, fine-needle aspiration biopsy^(1,13,14)^.

**Figure 1:**
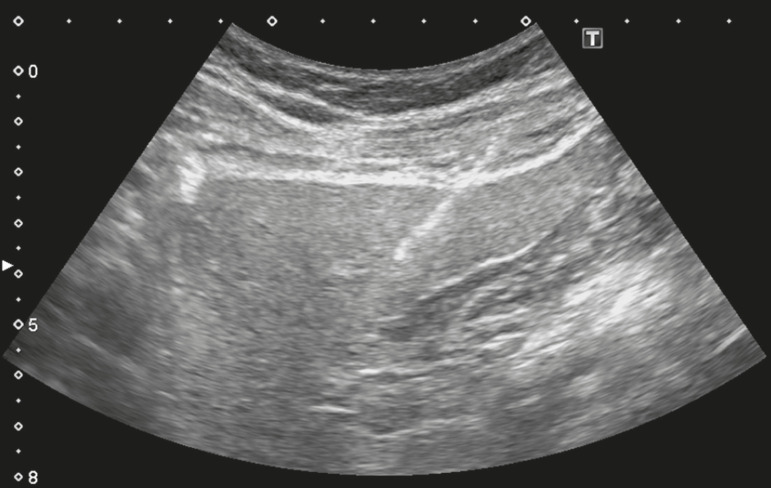
Sagittal view of a Tru-Cut biopsy of the left liver lobe under realtime ultrasound guidance after deployment of the needle. Note that the trajectory of the needle can be observed while it is being advanced through soft tissues.

The use of imaging guidance has improved the accuracy of percutaneous procedures and decreased complication rates^(3,14)^. Although a variety of imaging guidance methods can be used, the most popular methods are ultrasound and computed tomography (CT), the former, as depicted in Figure 1, being used in over 96% of procedures^(12,15)^. Ultrasound is the preferred technique because of its wide availability, ease of use, and flexibility of scanning planes, as well as because it does not expose patients to ionizing radiation^(15)^. In most patients, the liver is easily accessible through ultrasound. It is also a real-time technique, allowing the needle path to be visualized at all times during the procedure^(3,16,17)^.

During ultrasound-guided puncture, the needle is usually inserted by using an in-plane approach, in order to visualize the entirety of the needle, including its tip. The in-plane approach is preferable to an out-of-plane approach, in which the needle is observed in cross-section, the disadvantage being that the needle is crossed only once by the ultrasound beam, which can lead to misinterpretations of the needle tip position^(18)^. An additional variation is the use of a guidance device attached to the transducer, to secure the needle and guide it in a predetermined direction. However, those devices are costly, and, once the needle has been secured to the device, the angles and approaches are static, no repositioning being possible^(19-21)^. In most cases, punctures are performed with a free-hand technique, allowing virtually infinite planes and approach angles, as well as allowing changes in those during the procedure^(21)^.

Complications after ultrasound-guided percutaneous liver biopsy are rare, the most common being pain (with or without the need for analgesia), followed by, at a much lower incidence, hematoma, pneumothorax, and hemobilia^(3,11,15,22,23)^. For diffuse liver diseases and multilobar nodular diseases, such as metastases, the laterality of the specimen collection is at the discretion of the operator. Right lobe biopsy, through an intercostal approach, is preferred by most of the operators. On the other hand, most radiologists prefer a left lobe subxiphoid route^(13)^. It has been hypothesized that a right liver path would result in more postprocedural pain because it passes through diaphragmatic and intercostal muscle fibers^(24)^.

Although postprocedural patient comfort is a key objective when percutaneous liver biopsy is performed on an outpatient basis, the approach to using prophylactic pain medication in biopsies in general is controversial. In most cases, premedication is withheld, despite evidence that preprocedural sedation and analgesia has beneficial effects on patient anxiety and post-biopsy pain^(25)^. In real-life settings, most liver biopsies continue to be performed under local anesthesia^(13,15,23)^.

To standardize the evaluation of pain provoked by interventional procedures, a visual analog scale (VAS) is commonly used in order to provide a subjective assessment of pain by the patient^(25)^. The VAS pain score is derived from subjective self-report measurement of symptoms, translated from a handwritten mark placed at one point along a 10-cm line. The line represents a continuum from the absence of pain, on the left (at 0 cm), to the “worst possible pain” on the right (at 10 cm). The marks are translated in centimeters for record and can be tracked for pain evolution or comparison between patients^(26)^.

Given the low incidence of adverse effects, a large number of patients are needed in order to achieve adequate statistical power. The objective of this study was to evaluate differences in the need for analgesia after ultrasound-guided percutaneous liver biopsy performed on an outpatient basis in unsedated patients, analyzing variations regarding age, sex, laterality of the specimen collection, and indication for the biopsy (nodular vs. diffuse disease).

## MATERIALS AND METHODS

This was a retrospective cross-sectional study involving the analysis of data related to outpatient liver biopsies performed at our institution between 2012 and 2018. Data were retrieved from the procedure database of the institution, which includes data on clinical indication, puncture laterality (right vs. left lobe), number of liver punctures, needle size and throw, picture archiving and communication system image registration of biopsy tract, and clinical data relevant to the postprocedural observation period. The inclusion criteria were being ≥ 18 years of age; having been referred to our tertiary care center for outpatient liver biopsy because of nodular or diffuse disease; having received no sedation or intravenous anesthesia during the procedure; having been submitted to a single liver puncture with an 18G Tru-Cut needle with a 2.0-cm throw; and a single fragment having been retracted during the biopsy. The procedures were performed by four different radiologists with at least three years of experience in interventional procedures and were analyzed by one dedicated pathologist with 11 years of experience in 2012. Repeat procedures performed in the same patient on different dates were considered new events. The study was approved by the local research ethics committee (Reference no. 2.308.943). Because of the retrospective nature of the study, the requirement for written informed consent was waived.

The biopsy prescription written by the treating physician, consisting of a brief medical history and a statement regarding the indication, was analyzed by one of the staff radiologists. Administrative personnel contacted the patient by telephone to check for recent coagulation studies and complete blood count, as well as to provide the patient with instructions regarding preparation. Strict coagulation parameters were followed, which means that we scheduled only patients who were not under anticoagulant therapy or that were able to withhold anticoagulation, with an international normalized ratio ≤ 1.5, a platelet count > 50,000 cells/mm^3^, and an activated partial thromboplastin time < 1.5 times greater than normal, in the last four weeks. Patients were instructed to fast for 6 h before the procedure, which was scheduled in the next available half-hour time slot, from 7:00 a.m. to 3:00 p.m. Before each procedure, the performing physician informed the patient of the risks, benefits, and success rates associated with the procedures. Baseline vital signs (blood pressure, heart rate, and oxygen saturation) were assessed by the staff nurse.

### Procedure

All procedures were performed under real-time guidance with the same ultrasound system (Aplio 300; Toshiba Medical Systems, Tokyo, Japan) with 1.9-6.0 MHz convex array transducers (Toshiba Medical Systems), under local anesthesia from the skin to the liver capsule with 10 mL of 1% lidocaine, and involved the use of 18G core biopsy needles with a 2.0-cm throw-either automatic (Bard Magnum; C.R. Bard, Inc., Covington, GA, USA) or semi-automatic (SuperCore Semi-Automatic Biopsy Instrument; Argon Medical Devices, Frisco, TX, USA). For patients with diffuse diseases such as steatosis and cirrhosis, the liver lobe to be targeted was determined by the physician. For patients with multifocal lesions, the most accessible lesion with adequate parenchymal layering was targeted. For patients with solitary lesions, the laterality was naturally determined by that of the lesion side. In right lobe punctures, the standard approach was intercostal, whereas a subxiphoid approach was used for left lobe procedures. We selected only those procedures in which the liver capsule was punctured only once and no coaxial system was used.

### Need for analgesia

For the first 3 h in the recovery room, patients who had undergone a left lobe puncture were required to remain in the supine position, whereas those who had undergone a right lobe puncture were required to remain in the right lateral decubitus. For the next hour, all of the patients were sitting. Routine evaluations in the recovery room were performed by a nurse immediately after the procedure, as well as at 15 min, 30 min, 45 min, 1 h, 2 h, 3 h, and 4 h after the procedure, unless a complication was detected. In each evaluation, patients were questioned about pain (yes or no). If they answered yes, a radiologist was called for a more detailed evaluation, in which pain was graded with a visual analog scale. Physicians would reassure and re-evaluate patients with mild pain (1-3 on the VAS), prescribe analgesics for those with moderate pain (4-6 on the VAS), and prescribe opioids for those with severe pain (7-10 on the VAS). The analgesics used were either dipyrone (1 g) or acetaminophen (750 mg). When opioids were required in order to manage the pain, morphine was prescribed, at a dose of 4 mg (for patients < 70 years of age) or 2 mg (for patients ≥ 70 years of age). At the end of the 4-h observation period, patients with no complications were discharged and those with pain or any complication were held for further observation. If a patient reported persistent or increasing pain after analgesia, if the physical examination revealed signs of intra-abdominal bleeding (i.e., peritoneal irritation), or if there were changes in the vital signs suggestive of acute bleeding (e.g., increased hearth rate and hypotension), CT angiography would be promptly performed and analyzed by the radiologist, who would prescribe the appropriate treatment.

### Statistical analysis

Data are presented as mean ± standard deviation or as absolute and relative frequencies. We used the chi-square test to quantify associations between variables. Values of *p* < 0.05 were considered statistically significant. All statistical analyses were performed using the Predictive Analytics Software package, v. 18.0 (SPSS Inc., Chicago, IL, USA).

## RESULTS

**Figure 2:**
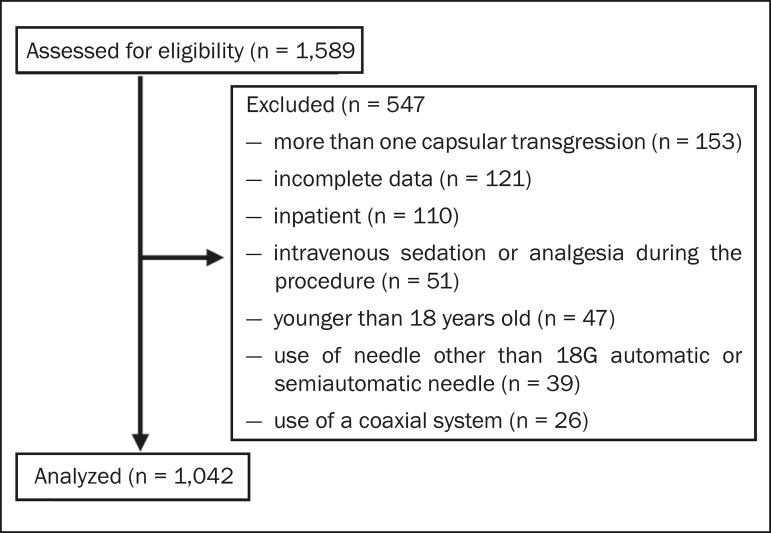
Flow chart of the selection process.

Between 2012 and 2018, a total of 1,589 liver biopsies were performed at our institution (Table 1). Of those, 1,042 met the study inclusion criteria, 485 having been performed in females and 557 having been performed in males (Figure 2). Patient ages ranged from 18 to 82 years, the mean age being 52.5 years. The main indications for biopsy were related to diffuse disease (in 89.9%), including the follow-up of hepatitis C (in 47.0%) and suspicion of nonalcoholic steatohepatitis (in 38.0%). Among the biopsies for which the indication was a nodular lesion (10.1%) the most common indication was suspicion of colorectal cancer metastases (in 3.3%). The right lobe was targeted in 69.6% of the cases. Nodular lesions were sampled in 13.7% of the left lobe biopsies and in 8.5% of the right lobe biopsies (*p* < 0.05).

**Table 1:** Patient population characteristics.

Parameter	(N = 1,042)
Sex, n (%)	
Male	557 (53.4)
Female	485 (46.6)
Referred for suspicion or follow-up of diffuse disease	937 (89.9)
Hepatitis C follow-up	490 (47.0)
Suspicion of nonalcoholic steatohepatitis	396 (38.0)
Other	51 (4.9)
Referred for nodular lesion biopsy	105 (10.1)
Suspicion of colorectal cancer metastases	35 (3.3)
Other	70 (6.7)
Lobe punctured	
Right	725 (69.6)
Left	317 (30.4)

During recovery room follow-up, 8% of the patients reported pain strong enough to require supplementary analgesia (> 3 on the VAS). Analgesia was required by 7.7% of the patients who had undergone a right lobe puncture and by 8.9% of those who had undergone a left lobe puncture, no statistical difference being found between the two groups. Of the 485 female patients, 51 (10.5%) reported pain requiring analgesia, compared with 33 (5.9%) of the 557 male patients (*p* < 0.05). The need for analgesia did not differ by patient age or in relation to the indication for biopsy (nodular vs. diffuse disease). The analgesic most commonly used was dipyrone (in 75.9%), followed by paracetamol alone (in 16.4%) and either in combination with an opioid (in 7.6%).

All but one patient was discharged after the 4-h observation period. In one patient, who had been referred because of suspicion of multifocal liver metastases, CT angiography showed an expanding hematoma with active bleeding after a right liver puncture. That patient was treated with superselective embolization and was discharged after a 48-h observation period in the ward.

## DISCUSSION

Despite the recent development of noninvasive methods for liver evaluation, liver biopsy continues to be the classical gold standard and an essential tool in the investigation and histological follow-up of liver diseases^(6,7,14)^. Liver biopsy can be performed through a myriad of different methods, including blind percutaneous and laparoscopic biopsy, as well as biopsy performed under the guidance of imaging modalities, which include fluoroscopy (transjugular liver biopsy), CT, magnetic resonance imaging, and ultrasound^(10,12,27-31)^.

Because of its wide availability, low cost, real-time imaging, and versatility of planes of approach, together with the fact that it does not expose patients to ionizing radiation, ultrasound is currently the most widely used method^(15)^. A series of different material sampling techniques can be used, including fine needle aspiration, the Menghini technique, and core biopsy^(13)^, the last being the preferred method. Using needles of at least 18G precludes the need for more experienced pathologists^(16)^. Augmenting diagnostic accuracy by retrieving more tissue specimens with larger bore needles comes at the cost of increasing the risk of complications^(23,32,33)^. The pathologists at our institution consider the use of 18G needles the best option for striking a balance between diagnostic accuracy and the risk of complications.

In the present study, the complication rate was particularly low, there having been just one major complication, defined as grade 3 in the Cardiovascular and Interventional Radiological Society of Europe Classification System for Complications, among the more than 1,000 cases evaluated. The low rate of complications, in comparison with that reported in other studies^(1,3,11,12)^, might be explained by the fact that we excluded procedures in which there was more than one capsular puncture or more than one tissue specimen was retrieved and included only procedures in which an 18G needle was employed. In addition, the study population was composed of patients considered normal within strict coagulation parameters. Although a major complication was a rare outcome in this study, we recommend that liver biopsies be performed in tertiary care centers, where such complications can be managed and treated promptly.

There has been debate regarding the need for periprocedural sedation and routine intravenous anesthesia during liver biopsies, although some institution use it on a routine basis^(25,34)^. At our institution, cooperative outpatient biopsies are performed using local anesthesia that consists of 10 mL of 1% lidocaine, without epinephrine, as is the case at most of health care facilities offering this kind of procedure.

In the present study, laterality did not prove to be an issue to be considered regarding post-biopsy pain. The need for analgesia was similar in the right and left lobe puncture groups, even after adjustment for all relevant variables. In addition, the need for analgesia was more common among the female patients than among the male patients. That might be because women have a different response to pain, have a different relationship with their body, or simply feel more comfortable expressing a need for analgesia to the nurses at our institution, the majority of whom were female.

None of the patients in our sample sought treatment for pain or complications after the 4-h observation period, indicating that it is a sufficient postprocedural observation period for liver biopsies. The one patient who developed a complication presented with increasing pain, within the first 30 min after the puncture, that did not respond to dipyrone or morphine. The complication was promptly diagnosed by CT angiography as an expanding hematoma and was treated by selective embolization.

Despite its significant statistical power, our study has limitations. One such limitation is the retrospective nature of the study. The fact that we included only outpatients, who are, in theory, more fit than are inpatients, might constitute a selection bias. In addition, to homogenize the sample, we did not include procedures with more than one capsular puncture, which could have increased the complication rate significantly.

## CONCLUSION

In conclusion, ultrasound-guided percutaneous liver biopsy is a procedure that is safe and well tolerated. The most common complication is pain, which seems to be intrinsically related to the procedure itself, not correlating with the laterality of specimen collection, patient age, or the type of disease (nodular vs. diffuse). An observation period of 4 h appears to be sufficient to detect virtually all complications.
